# Impact of Chronic HIV/SIV Infection on T Follicular Helper Cell Subsets and Germinal Center Homeostasis

**DOI:** 10.3389/fimmu.2016.00501

**Published:** 2016-11-11

**Authors:** Stéphanie Graff-Dubois, Angeline Rouers, Arnaud Moris

**Affiliations:** ^1^Sorbonne Universités, UPMC Univ Paris 06, INSERM, Centre d’Immunologie et des Maladies Infectieuses, U1135, CNRS 8255, Paris, France

**Keywords:** HIV, SIV, Tfh cell differentiation, Tfh cell dynamics, germinal center reaction

## Abstract

The discovery of broad and potent HIV-1 neutralizing antibodies (bNAbs) has renewed optimism for developing an effective vaccine against HIV-1. The generation of most bNAbs requires multiple rounds of B cell receptor affinity maturation, suggesting a crucial role of follicular helper T (Tfh) cells in their production. However, less than 1% of HIV-infected patients develop bNAbs that arise late in the course of infection, indicating probable Tfh and B cell dysfunctions in this context. Since the last few years, many studies have characterized Tfh cells from lymph nodes and spleen of HIV-infected individuals and SIV-infected macaques. Various lymphoid Tfh cell subsets have been identified, including precursor Tfh (pTfh), germinal center Tfh (GC Tfh), and the regulatory counterpart of Tfh cells, the follicular regulatory T cells. The latter have been reported to play a crucial role in the control of T and B cell crosstalk and GC reactions. More recently, circulating Tfh-like cells (cTfh) have been identified. Meanwhile, advances in single-cell technologies have made possible to analyze the transcriptional profiles of low abundant cells, such as Tfh populations. Using transcriptional signatures, we review here the impact of chronic SIV/HIV infection on Tfh, GC Tfh, pTfh, and cTfh differentiation and helper T cell functions with regard to their capacity to induce efficient B cell maturation. We will explore some hypothesis to explain the increased proportion of Tfh cells reported in chronically infected individuals and the impact on HIV pathogenesis.

## Introduction

In germinal centers (GC), T follicular helper (Tfh) cells deliver helper signals and cytokines required for B cell affinity maturation and B cell differentiation into long-lived plasma cells. Optimal Tfh and B cell crosstalk is a prerequisite for the induction of efficient humoral immunity to pathogens. By providing survival and differentiation signals, Tfh cells control multiple steps of B cell maturation and antibody (Ab) production.

In addition to the cognate antigen interaction with B cells, Tfh cells express costimulatory molecules, such as CD40L, ICOS, and OX40. Tfh cells secrete high levels of interleukin-21 (IL-21) and IL-4, which are necessary for GC formation and B cell differentiation into long-lived plasma cells, respectively (1–3).

Among tissue-resident Tfh cell subsets, early committed precursor Tfh (pTfh) and germinal center Tfh (GC Tfh) represent two different stages of the Tfh cell maturation. Follicular regulatory T (Tfr) cells are identified as the regulatory counterpart of Tfh cells. Tfr cells control T and B cell crosstalk and GC reactions. Blood circulating Tfh cells (cTfhs) have been recently identified as a memory compartment of tissue-resident Tfh cells. Like tissue-resident Tfh cells, cTfhs are endowed with the capacity to produce IL-21 and to provide B cell help (4).

Since the last 5 years, Tfh cells have been extensively studied in the lymph nodes (LNs) and spleens of individuals with chronic HIV/SIV infection. HIV infection is associated with an altered B cell differentiation (5) and Tfh isolated from LNs of HIV-infected (HIV+) individuals provide inadequate B cell help *in vitro* (6). As lymphoid tissue-resident Tfh cells are targeted by HIV/SIV early after infection, they constitute a major compartment for HIV infection, replication, and production of viral particles in LNs of viremic individuals (7–9), even though *in vivo* production of viral particles by Tfh cells remains to be demonstrated. Likewise, in blood, within central memory CD4T cells, cTfh cells serve as HIV reservoir in chronic HIV-infected individuals under antiretroviral therapy (10). Very recently, in natural HIV controllers, study of HIV infection in various CD4 T cell subsets demonstrates various mechanism of HIV persistence according to the CD4 T cell compartment (11). LN-resident helper T cells (Tfh and non-Tfh) showed replicative virus, while clonally expanded blood CD4 T cells harbor inducible provirus (11). However, despite their high susceptibility to HIV/SIV infection, many studies reported an accumulation of tissue-resident or cTfh populations during the chronic phases of infection (7, 8, 12, 13). In addition, Hong et al. demonstrate that after a rapid expansion of GCs during the acute phase, slowly proliferative Tfh cells accumulate during the chronic phase of SIV infection (14).

Various hypotheses can support the higher proportions of Tfh cell subsets in the context of chronic HIV infection: (i) Tfh cells might present high proliferative or survival capacities; (ii) antigen persistence could drive CD4 T cells toward Tfh differentiation; and (iii) regulatory cells that control the Tfh/B cell crosstalk might be defective.

Here, we propose to review recent studies based on transcriptional analysis of Tfh cell subsets and to discuss the potential consequences on GC deregulations reported in chronic HIV/SIV infection.

## Potential Impact of HIV Infection on Tfh Cell Differentiation

**Figure 1:**
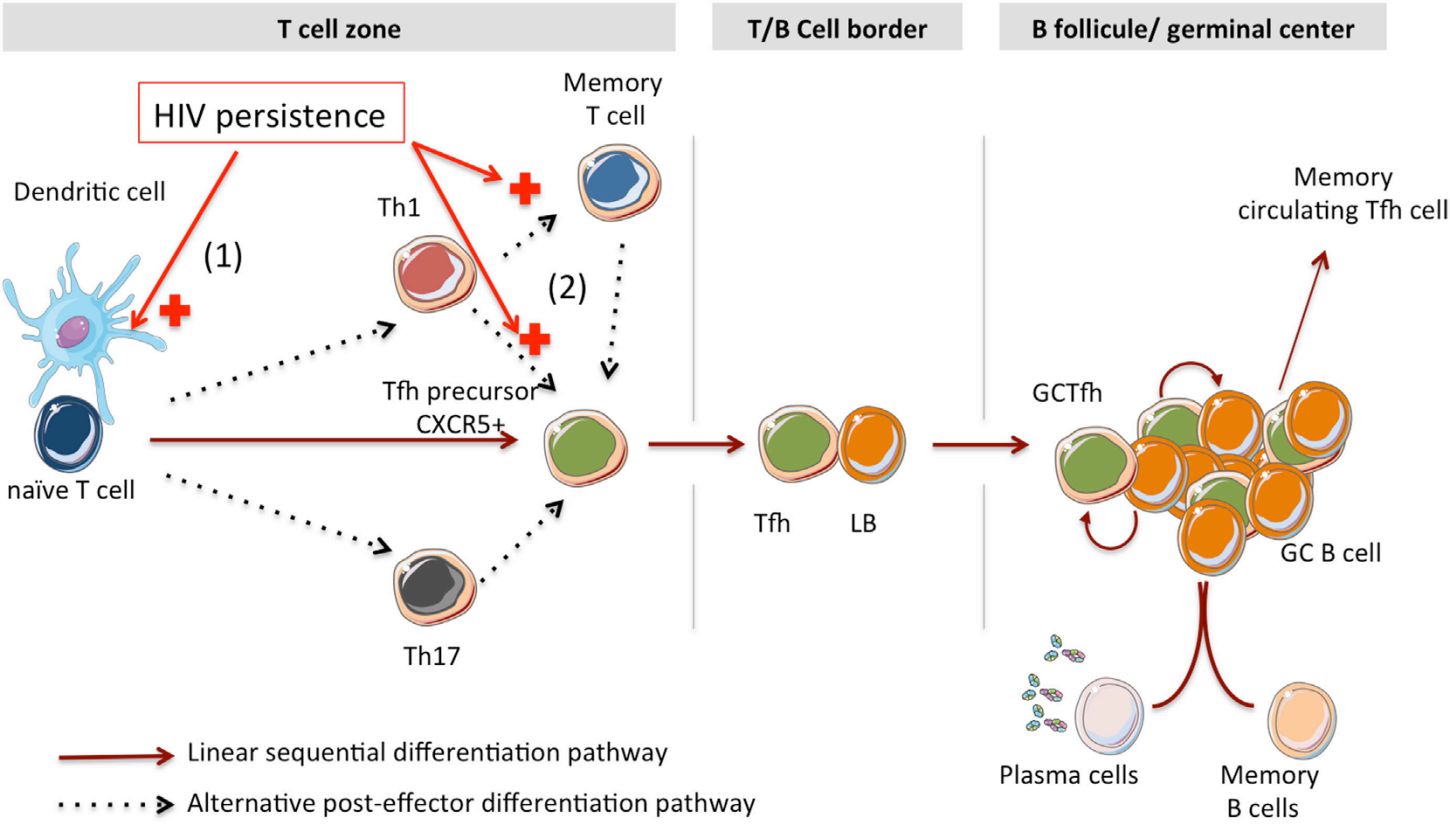
**Impact of HIV antigen persistence on Tfh cell differentiation in lymphoid tissues**. Linear and alternative post-effector Tfh cell differentiation pathways are described. (1) Interactions between HIV particles and DC-SIGN expressing DCs could support the T helper cell differentiation toward a Tfh polarization. (2) According to the alternative post-effector differentiation pathway, HIV persistence might support Th1 and memory T cell differentiation into Tfh cells.

The signals involved in Tfh cell differentiation include TCR activation, costimulation, cytokines, and migration-associated molecules. However, the origin of Tfh cells is not well defined in humans: it is not clear whether Tfh fate is established at the time of DC priming or later. Here, we review the impact of HIV infection on Tfh cell differentiation, from the priming of CD4 T cells by DCs cells until their ultimate stage of differentiation corresponding to GC Tfh and circulating memory Tfh. Two distinct differentiation pathways have been described (Figure 1).

The linear multistage Tfh differentiation pathway implicates multiple antigen-specific interactions in secondary lymphoid organs: (i) DC priming of naïve T cells leads to the rise of pTfh cells expressing CXCR5 molecule; pTfh cells migrate toward the T/B cell border zone where they experience (ii) a second antigen-specific interaction with B cells. This interaction leads to the progression of pTfh cells within the B cell follicle and differentiation into Tfh cells. (iii) In the B cell follicle, Tfh cells experience multiple interactions with B cells, leading to B cell maturation and the complete differentiation of Tfh cells into GC Tfh. Thus in this model, B cells appear central in the terminal differentiation of Tfh cell into GC Tfh and reciprocally, Tfh are also required for B cell maturation. As with other helper T cell subsets, the stimulatory cytokines produced by DCs during the priming of naïve T cells are critical parameters of Tfh cell differentiation. Using monocyte-derived DCs (MoDCs), Schmitt et al. demonstrated the key role of IL-12-producing MoDCs in the induction of IL-21-producing Tfh-like cells (15). More recently, the same group found that TGF-β acts together with IL-12 and IL-23 to induce the expression of various molecules associated with Tfh functions by human naïve helper T cells, including CXCR5, ICOS, IL-21, Bcl-6, and the transcription factors BATF and c-Maf (16). However, little is known about the type of DCs responsible for inducing Tfh cell priming. A recent study reported that engagement of DC-SIGN by fucose-based PAMPs licenses DCs for inducing Tfh polarization (17). Such activated DCs produce IL-27, which is essential for Tfh polarization. This finding highlights the importance of adjuvants in the induction of Tfh cells. Interestingly, HIV particles bind DC-SIGN through Gp120, the viral envelope (18). Therefore, one can hypothesize that, under chronic HIV infection, interactions between HIV particles and DC-SIGN expressing DCs could support the T helper cell differentiation toward a Tfh polarization. A recent study also showed that treatment with CpG (TLR-9 ligand) induces IL-6 production by MoDC, orientating helper T cells differentiation toward the Tfh-cell lineage (19). Indeed, by inducing Bcl6 early during the T cell activation, IL-6 has been shown to be critic for Tfh polarization (20). In the context of HIV/SIV infection, several groups reported higher plasma levels of IL-6 (13, 21). However, we and others (22) did not find any difference in the amount of secreted IL-6 between HIV-infected and -uninfected spleens upon activation (8). Once engaged into Tfh differentiation, the sequential differentiation model proposes that pTfh/B cell interactions dictate the fate of Tfh cells. Several groups highlighted the requirement of antigen presentation by B cells to induce Tfh cell and in turn GC reactions. In the absence of B cells, DC restricted antigen presentation initiates Tfh cell differentiation (into pTfh) but fails to complete ultimate effector Tfh cell differentiation (23). In a model of bone marrow chimera, B cells deficient for the expression of MHC-II-molecules exhibit a reduced capacity to initiate T cell expansion and differentiation (24). In fact, sequential antigen-specific interactions of Tfh cell with DC and B cells are required to initiate Tfh cell and GC differentiation (25), and antigen persistence sustains Tfh responses and GC reactions (26). Using live multiphoton imaging, Schwickert et al. suggested that the amount of peptide–MHC (pMHC) complexes presented by antigen-specific B cells to cognate T cells, at the B-cell–T-cell border, was a limiting factor regulating the entry of B cell clones into GC (27). Furthermore, highlighting the critical role of MHC-II molecules expressed by B cells in the generation of Abs of diverse functions and of memory B-cell responses, B cells lacking MHC-II expression are unable to differentiate into memory cells and are defective in producing antigen-specific IgG (28). These results demonstrate that MHC-II-restricted antigen presentation by B cells is strictly required for B cells to receive help by antigen-specific Tfh cells, and thus to establish a potent humoral immune response. Therefore, Tfh cell differentiation and GC development require the combination of DC and B cell antigen presentation.

As DC during T cell priming, B cells also provide additional signals to Tfh cells, contributing to their helper functions and maintenance. These signals include CD40L/CD40, OX40/OX40L, signaling lymphocyte activation molecule (SLAM) family members, and adhesion molecules that strengthen GC Tfh/GC B cell interaction. Interaction between ICOS and ICOS ligand (ICOSL) as well as IL-21 production has been implicated in GC formation (29, 30). PD-1/PD-1 ligand interactions also control Tfh and GC B cell differentiation (1, 31). Murine Tfh cells also express the nutrient transporter folate R 4 (FR4) and CD73 (32) although their functional relevance for Tfh cell differentiation and B cell help has not yet been uncovered. In sum, the sequential differentiation proposes that combined interactions with DC and B cell dictate the fate of Tfh cells.

The alternative “post effector” developmental pathway proposes that Tfh-like cells may develop either from the memory CD4 T cell lineage (33, 34) or from effector T helper cell subsets (35, 36), rather than arising from pTfh cells. It has been shown that Tfh and central memory T cells (T_CM_) are similar in their developmental pathway, including the requirement of Bcl6 and low levels of IL-2 signaling (37). In line with this, Tfh and T_CM_ gene programs can co-initiate from effector Th1 cells upon increased Bcl-6 expression in response to a decrease of IL-2, resulting in a “Tfh/T_CM_-like” population. IL-7 signaling also acts as a negative feedback that downregulates the differentiation of Th1 into Tfh-like cells (38). Interestingly, in spleens from HIV-infected individuals with a high proportion of Tfh cells, we reported a markedly reduced expression of the IL-7r encoding gene in all CD4 T cell populations (8). Taken together these observations support the hypothesis that, in addition to Tfh cells, other T helper populations may contribute to B cell maturation into long-lived plasma cells. Hence, in humans, the precursors of Tfh cells might be composed of heterogeneous cell populations, which have the ability to differentiate into distinct types of Tfh cells. The latter keep some functional imprint from the parental T cell subtype. Of note, in this alternative pathway, interactions with antigen-presenting B cells are still a key event of the Tfh cell orientation thus raising the question of the impact of Tfh infection by HIV on the B cell compartments.

Germinal center Tfh cells have long been considered as the terminal stage of tissue-resident Tfh cell differentiation. Newly identified, memory Tfh cells are preferentially located in secondary lymphoid organs and bone marrow although they can recirculate in the blood. These cTfh involve several subsets that differentially support Ab secretion (4) and are related to lymphoid-tissue-resident Tfh cells by their gene expression profile, cytokine production, and functional properties (39). Recently, adding to the CXCR5 and PD-1 canonical markers, Schultz et al. proposed that cTfh can be identified by their ability to produce IL-21, the cardinal Tfh cytokine (40). Interestingly, activated memory B cells induce rapid re-expression of Bcl6 by memory Tfh cells (41), reinforcing the concept that many features of Tfh cells are highly linked with those of the B cells. Thanks to their accessibility and relative high frequencies, cTfh cell dynamics and features are the focus of growing interest in the context of infection and vaccination.

## The Frequency and Functions of Tfh Cells are Tightly Controlled

Follicular helper T cell homeostasis is critical to the induction of high affinity Ab responses that are devoid of self-reactivity. Indeed, optimal Tfh cell frequency imposes competition between B cells, thus favoring survival of high affinity B cell clones. Several cell populations maintain Tfh cell homeostasis, including regulatory T (Treg) cells, Tfr cells, CD8 regulatory cells, and plasma cells (42). Tfr cells are identified as the main T cell subset implicated in the control of Tfh cells. They migrate into follicles and directly control GC reaction (43, 44). Hence, many studies have demonstrated increased GC and T cell responses in the absence of Tfr (43, 45, 46). Tfr cells co-express Bcl6 and Blimp-1 (43) that is known to negatively regulate Tfh cell differentiation pathway (47). Indeed, Blimp-1 represses Bcl-6 and reciprocally, which might explain the lower expression of Bcl-6 in Tfr cells as compared to Tfh cells (43). Tfr cells express CTLA-4 and produce high amounts of IL-10. They have been shown to arise from Foxp3+ precursors that highjack the Tfh differentiation pathway. However, a recent study showed that, using an appropriate vaccine adjuvant, Tfr cells can derive directly from naïve CD4 T cells (48). Alteration of Tfr cells functionality might contribute to higher proportions of Tfh cells during HIV-infection. Our results indicated that HIV infection did not impact splenic Tfh/Tfr ratio suggesting that Tfr and Tfh cell subsets expended equally during HIV infection (8). However, Chowdhury et al. have shown a limited expansion of Tfr cells as compared to the one of Tfh cells during SIV infection (49). They explored the transcriptional profile of CXCR5+ PD1hiCD127-CD25+ Tfr cells after SIV infection. Overall, genes linked with Tfh differentiation and functions, such as PD-1, IL-6R, SLAMF6, and CD84, were more expressed in Tfr cells, while expression of IL-2RA linked with Treg functions was reduced after SIV infection suggesting that SIV infection might impair expression of genes associated with Treg and thus Tfr regulatory functions (49).

According to their transcription profile, Tfr cells are situated between Tfh and Treg cell subsets. However, foxp-3 expression is not taken into account in most Tfh cell studies that *de facto* include Tfr subset among Tfh cells. Recently, adding to Tfr cells, Treg cells expressing CTLA-4 have been reported as major inhibitors of B cell expression of CD80 and CD86, which are essential to the induction of Tfh cells (50–52).

## Tfh Cell Dynamics During the Course of HIV/SIV Infection

Follicular helper T and cTfh cells are targeted by HIV/SIV very early after infection and constitute a major compartment for HIV replication and production of viral particles in LNs and periphery of viremic individuals (7–9, 11). Despite their high susceptibility to HIV/SIV infection, most studies reported an accumulation of tissue-resident or cTfh cell populations (7, 8, 12, 13). Interestingly, the Tfh cell frequency positively correlates with plasma viremia levels (7, 12), and Tfh cell accumulation is reduced in individuals that control SIV infection (53), suggesting that the persistence of viral antigens might drive Tfh cell expansion. Accordingly, cTfh cell expansion has been recently reported in untreated individuals while the frequency of cTfh cells is restored to normal levels under cART suggesting that HIV replication also drives cTfh cell dynamics (10). Most studies report an increase of Tfh cells among memory CD4 T cells during HIV/SIV infection, whereas others conclude with the opposite statement (9, 54). In SIV-infected rhesus macaques, Moukambi et al. recently showed that Tfh dynamics differs from one compartment to another (peripheral blood vs. LNs or spleen) (9). Moreover, the Tfh cell frequency varies according to (i) the stage of HIV/SIV infection (53), (ii) the severity of the disease, and (iii) the ability to develop broadly neutralizing antibodies (bNAbs) (39, 55). In Table 1, we summarized Tfh cell dynamics from various studies taking into account: the type of infection (HIV/SIV), the phase (acute vs. chronic), the disease progression (slow vs. fast), the immune compartment (peripheral blood vs. secondary lymphoid organs), the phenotype, and the antigen specificity of the Tfh cells. Irrespective of the immune compartment (LNs, spleen, or blood), Tfh cells are preserved in HIV/SIV controllers, displaying no Tfh cell accumulation or loss. On the contrary, Tfh cell loss is reported in fast progressors as well as in the late stages of disease.

**Table 1:** **Tfh cell dynamics in HIV/SIV infection according to the stage (acute, chronic, or late) and the outcome of the disease**.

	Phase	Disease outcome	Compartment	Phenotype	Antigen specificity	Dynamics	Reference
SIV	Acute	P	Spleen	CXCR5+PD-1+	Total	Loss	(9)
SIV	Acute	Slow P	LN	CXCR5+PD-1+	Total	Accumulation	(14)
SIV	Acute	Fast P	LN	CXCR5+PD-1+	Total	No Accumulation	(14)
SIV	Chronic	Slow P	LN	CXCR5+PD-1+	Total	Accumulation	(56)
SIV	Chronic	Slow P	Spleen, LN	CD45RA-CD62L+CXCR5+PD-1+	Total	Accumulation	(9)
SIV	Chronic	Fast P	LN	CXCR5+PD-1+	Total	Loss	(9)
SIV	Late	P	LN	CXCR5+PD-1+	Total	Loss	(53)
SIV	Chronic	C	LN	CXCR5+PD-1+	Total	Preservation	(53)
SIV	Chronic	P	LN	CXCR5+PD-1+	Total	Accumulation	(57)
SIV	Chronic	ND	LN	CD28hiCD95hi CCR7loPD-1hi	Total	Accumulation	(13)
HIV	Acute	C	Blood	CXCR5+PD-1+	Total	Preservation	(55)
HIV	Chronic	P	Blood	CCR7+CXCR5+CCR6+PD-1+(±CXCR3)	Total	Loss	(54)
HIV	Chronic	High neutralizers	Blood	CCR7lowCXCR5+PD-1+CXCR3-	Total	Preservation	(39)
HIV	Chronic	ND	Blood	CCR7+CXCR5+PD-1+CXCR3−	Total	Accumulation	(10)
HIV	Chronic	ND	LN	CXCR5+PD-1+Bcl-6+	HIV-specific	Accumulation	(7)
HIV	Chronic	ND	LN	CXCR5+PD-1+Bcl-6+(CCR7−CD45RA−)	Total/HIV-specific	Accumulation	(12)
HIV	Chronic	ND	Spleen	CCR7-CD45RA-CXCR5+PD-1+	Total	Accumulation	(8)

*P, progressors; C, controllers; ND, non-determined*.

*The phenotype and the immune compartment [spleen, blood, or lymph nodes (LNs)] are mentioned*.

Follicular helper T cell accumulation is reported during slow progression (SP) or chronic stage of the disease. Indeed, evidences support the pivotal role of persistent viral antigen within the GC in driving Tfh cell expansion. HIV particles are associated with FDC in tonsils and LNs from infected patients (58–60) and Cheynier et al. reported the persistence of high levels of HIV particles in GC of HIV+ spleens from untreated subjects (61). In addition to FDC-bound virions, opsonized HIV particles interact with B cells trough CD21 membrane receptor (62, 63). Remarkably, the accessibility of CTL to GC is reduced, thus limiting the elimination of HIV-infected cells (64). Therefore, B cell follicles locally concentrate cell subsets implicated in HIV replication and viral production, which maintain antigen persistence and GC reactions. It appears that antigen persistence sustains ongoing GC reactions in which Tfh and GC B cell frequencies are highly correlated (8, 12, 65). However, a limited number of fully functional Tfh cells is required for the induction of bNAbs (42).

Another clue supporting the pivotal role of GC in driving Tfh cell expansion is that the disruption of GC organization coincides with the loss of Tfh cells and the onset of AIDS in terminal stages of SIV infection (53). PD1/PD-L2 axis contributes to the survival of Tfh and B cells. Interestingly, the expression of PD-L2 on B cells is severely impacted in the late stages of SIV infection potentially contributing to a decreased survival of T and B cells and the termination of GC reaction (53).

In sum, increased proportions of Tfh cells do not necessarily results in a better immune control of HIV infection, and Tfh cell proportions must be tightly regulated to allow efficient maturation and selection of B cells displaying high B cell receptor (BCR) affinity. Tfh cell functions need to be preserved to allow the production of potent bNAbs.

## Alteration of Tfh Cell Functions in the Context of HIV Infection

The key role of Tfh cells is to provide B cell helper signals and to promote their differentiation into memory B cell displaying high affinity for pathogens. These signals consist of production of cytokines, such as IL-4 and IL-21, and the expression of cell surface molecules, such as OX40, ICOS, and CD40L, by Tfh cells (1). As other groups, we analyzed the transcriptome profiles of Tfh as a mean to assess potential Tfh dysfunctions (see references in Table 2). Using single-cell sorting and high-throughput PCR (Fluidigm BioMark HD), we showed that expression of genes implicated in splenic Tfh and GC Tfh cell functions are deeply impacted by chronic HIV infection (8). In this section, we intend to review the impact of HIV/SIV infection on the main signals implicated in Tfh cell functions.

**Table 2:** **Transcriptional profiles of Tfh cells in HIV/SIV-infection**.

		Compartment	Population	Method	Helper functions						Differentiation				Regulation				Reference
					IL-21	IL-4	OX40	CD40L	ICOS	BCL6	CXCR5	MAF	CXCL13	STAT3	BLIMP1	PD-1	IL-10	CTLA4	
**HIV/SIV**+ **vs. HIV/SIV**^−^		Lymphoid organs	Tfh CXCR5+PD1bright	OR	=														(9)
			CD28hiCD95hiCCR7lo	OR		−							+						(13)
			PD1hiICOShiCD150lo																
			CCR7loCXCR5+PD1+	SC	+		−	−	−ns	+	=		+	−		−	−	−	(8)
		Blood	CXCR5hiCCR6hiPD1hi	OR	−	−			+										(54)
**Disease outcome**	**SP**	Lymphoid organs	Total splenocytes	OR						=	+	+							(9)
	**Neutra**		CD28+CD95+CXCR5+PD1hi CD154+	OR	+					+	+	+	+		−				(65)
	**C**	Blood	CD45RA-CXCR5+CXCR3−	SC	+					+									(66)

*Gene expression profiles presented by functions: helper, differentiation, migration, or regulation. Top of the table: differential expression of genes in HIV- or SIV-infected individuals as compared to HIV- or SIV-negative individuals. Bottom of the table: differential expression of genes depending on disease outcome. SP, slow progression, individuals presenting slow progressions (SP), high neutralization titers (Neutra), or HIV-control (C) are compared to individuals with fast progression, poor neutralization, and absence of HIV control, respectively*.

*Method: OR, overall reaction; SC, single-cell analysis*.

*+, higher expression; −, lower expressed; =, similarly expression; ns, non-significant*.

CD40L–CD40 (expressed by Tfh and B cell, respectively) interactions are required for the induction and maintenance of GC reaction. Blocking this molecular axis leads to GC disruption (67). In line with this, mutation in the CD40L gene is responsible for the X-linked hyper-IgM syndrome in humans characterized by a markedly decreased serum concentrations of IgA, IgE, and IgG (68). Our transcriptional data showed that the expression of CD40L gene is severely impacted in Tfh and GC Tfh from HIV-infected spleens (8). Of note, CD40L down modulation has been reported in global CD4+ T cell population during the late stages of HIV infection (69) and our unpublished data.

OX40–OX40L interaction is required for B cell differentiation into plasma cells (70). In humans, mutations in OX40 gene lead to decreased proportion of circulating memory B cells but do not impact the Ab responses (71). HIV-infected spleens exhibit defective expression of gene encoding OX40 in Tfh and GC Tfh cells (8). Intriguingly, we reported a reduction of memory B cell compartment in chronically HIV-infected individuals. Whether OX40 defective expression by Tfh is involved in this decrease in memory B cell should be further investigated.

During GC reactions, the expression ICOS by Tfh cells plays a major role in the process of selection of high affinity B cells. ICOS ligation leads to the overexpression of CD40L by Tfh cells that, in turn, promote the expression of ICOSL by GC B cells (72). In mice, recent findings emphasize the crucial role of T and B cell interactions through ICOS–ICOSL and CD40L–CD40 molecular axis in the maintenance of GC reactions and the production of high affinity bone-marrow plasma cells. ICOSL has been identified as a key regulator of positive selection of high affinity B cells during T–B cell interaction. Noteworthy, in comparison with uninfected donors, ICOS expression is enhanced in cTfh of ART-treated HIV-infected individuals (54) suggesting an overall immune activation of cTfh in those patients.

IL-21 is considered as the cardinal cytokine of the Tfh cell population. Tfh-secreted IL-21 induces B cell affinity maturation (73). A defective production of IL-21 by Tfh cells as well as a defective expression of its receptor by B cells severely impacts B cell proliferation and their differentiation into plasma cells (74). In the context of HIV infection, circulating CD4+ T cells secreting IL-21 are defined as the closest relative of tissue-resident (from secondary lymphoid organs) Tfh cells, both phenotypically and transcriptionally (40). cTfh cells from chronically HIV-infected individuals present altered expression of IL-21 gene (54) suggesting defective helper function. Conversely, we showed higher level of IL-21 transcripts in Tfh cells from chronically HIV-infected spleens (8) while Moukambi et al. reported a similar level of expression between Tfh from uninfected macaques and SIV-infected macaques during the early and the chronic phases of SIV infection (9). Discrepancies concerning the stage of the disease as well as the immune compartment (blood, spleen, and LNs) might explain these conflicting observations. Higher levels of IL-21 transcripts in cTfh cells are associated with HIV-controller status (66) or with cross-reactive neutralizing responses in rhesus macaques during the chronic phase of SIV infection (34–50 weeks post infection) (65).

IL-4 plays a major role in Tfh function (1) and confers anti-apoptotic properties to GC B cells that are particularly susceptible to cell death by apoptosis (75). IL-4 also participates to GC B cells selection (76). Tfh cells from SIV-infected rhesus macaques showed a marked reduction of IL-4 gene expression, and a global alteration of genes implicated in the network associated with IL-4 pathway (13). A defective expression of IL-4 gene has also been reported in cTfh from ART-treated HIV-infected individuals (54). We have shown a decreased secretion of IL-4 and IL-10 in splenocytes from chronically HIV-infected individuals (8). IL-10 contributes to B cell differentiation (77). Altered IL-10 secretion was also confirmed at the transcriptional level (8). However, our experimental settings did not allow the discrimination of Tfh cells from the Tfr subset and IL-10 deficiencies might also be related to defective Tfr functions.

SLAMF1 is specifically required for IL-4 and IL-21 production by GC Tfh cells (78), and SAP (SLAM-associated protein) is essential for GC reaction by stabilizing interaction between Tfh and B cells (79). In spleens from HIV-infected donors, we also observed a defective SLAMF1 gene expression in Tfh cell subsets that might explain the low level of IL-4 production by splenocytes from HIV-infected patients (8).

Altogether, these results indicate that HIV infection severely impacts Tfh cell functions in their capacity to provide adequate B cell help. These alterations result from both intrinsic Tfh cell defects and higher proportions of Tfh cells. Whether HIV infection alters Tfr cell functions is not clear. This issue will require further studies, especially addressing the capacities of Tfr cells to suppress Tfh cell functions or differentiation as well as B cell maturation. However, it will require identifying Tfr specific extracellular markers.

## Requirement for Early ART Initiation and Optimized Accessibility to Lymphoid Tissues

HIV/SIV infection results in antigen persistence that drives CD4 T cell differentiation toward the Tfh phenotype, thus maintaining high level of Tfh cells in GC. By controlling viremia, introduction of cART allows a normalization of cTfh cell proportions while Tfh cell functions are not fully restored. In particular, *in vitro* production of immunoglobulin is reduced in both untreated patients and HIV-infected individuals under cART (54). Hence, despite cART, HIV patients showing a detectable viral load (163 ± 178 HIV RNA copies/mL) did not respond to the 2009 H1N1/09 vaccine, whereas vaccine responders displayed undetectable viral loads (<40 HIV RNA copies/mL). This suggests that the persistence of viremia compromises Tfh cell functions (80). Altogether, early control of viremia appears crucial to preserve Tfh cells functions. Additionally, while B cell compartment is not recovered in chronically infected individuals under cART, early cART initiation restores the memory B cell compartment during acute HIV infection (81).

Early cART initiation during primo infection allows a dramatic decrease of cell-associated HIV DNA and thus limits establishment of the reservoir (82, 83). However, the poor penetration of cART into lymphoid tissues (84) could limit the efficiency of drugs even if cART was initiated early. To address these limitations, several approaches, mainly based on the molecular formulation of drugs, are actually envisaged. Injectable cART could overcome the limited access to lymphoid tissues. In the LATTE clinical trial, injection of combined cabotegravir and rilpivirine was well tolerated and efficient (85). However, the relevance of this approach on the persistence of the HIV reservoir has not been reported yet. Very recently, the efficient elimination of latently HIV-infected cells using HIV protease-sensitive toxin nanocapsules has been reported (86). This strategy presents the advantage to specifically eliminate HIV-infected cells without impacting healthy cells, allowing a less invasive approach. Combining new ART formulations with innovative route of administration could contribute to eradicate HIV reservoir from lymphoid organs.

In conclusion, HIV/SIV infections target Tfh cell subsets and severely affect Tfh cell frequency and functions, with dramatic impact on GC homeostasis. While limited number of fully functional Tfh cells is required for the induction of bNAbs, it is now well established that viral antigen persistence drives increased Tfh cell differentiation. To this regard, blocking HIV replication in lymphoid tissues might be a prerequisite to the induction of potent bNAbs. Preventing virus from entering lymphoid tissues should give another benefit to early ART initiation as well as the development of new strategies to optimize the access of ART to lymphoid tissues.

## Author Contributions

SG-D, AR, and AM wrote and approved the version to be published.

## Conflict of Interest Statement

The authors declare that the research was conducted in the absence of any commercial or financial relationships that could be construed as a potential conflict of interest.
